# An MRI based histogram oriented gradient and deep learning approach for accurate classification of mild cognitive impairment and Alzheimer’s disease

**DOI:** 10.3389/fmed.2025.1529761

**Published:** 2025-09-09

**Authors:** Arunnehru J., Shakir Khan, Anwar Basha H., Abdullah M. Albarrak, Aleem Ali

**Affiliations:** ^1^Department of Computer Science and Engineering (Emerging Technologies), SRM Institute of Science and Technology, Vadapalani, Chennai, Tamil Nadu, India; ^2^College of Computer and Information Sciences, Imam Mohammad Ibn Saud Islamic University (IMSIU), Riyadh, Saudi Arabia; ^3^Department of Computer Science and Engineering, Rajalakshmi Institute of Technology, Chennai, Tamil Nadu, India; ^4^Department of Computer Science and Engineering, Chandigarh University, Mohali, Punjab, India

**Keywords:** histogram oriented gradients, Harris corner, Alzheimers’s disease, mild cognitive impairment, deep neural network

## Abstract

Alzheimer’s disease (AD) is a common form of dementia that affects the central nervous system, causing progressive cognitive decline, particularly in memory. Early, non-invasive diagnosis is critical for improving patient care and treatment outcomes. This study proposes a robust feature extraction approach combined with three classifiers to achieve optimal classification of AD stages. T1-weighted brain MRI scans were used as input data. Features were extracted using Harris Corner interest points and the Histogram of Oriented Gradients (HOG) method. Classification was performed using Support Vector Machine (SVM), K-Nearest Neighbor (KNN), and a Deep Neural Network (DNN)-based pipeline. The proposed system classified three AD stages—Control Normal (CN), Mild Cognitive Impairment (MCI), and AD—with high accuracy: KNN (88%), SVM (91.5%), and DNN (95.6%). The DNN approach outperformed other classifiers and was further compared with state-of-the-art deep learning models, demonstrating competitive performance. These results highlight the potential of the proposed framework for early, accurate AD diagnosis using non-invasive imaging.

## 1 Introduction

Alzheimer’s is regarded as one of the primary causes of mortality. AD is the most prevalent form of cognitive impairment, comprising 50–80% of all cases. AD is characterized by a decline in cognitive performance that exceeds the normal age-related decline. Adverse effects are observed on memory, thinking, orientation, comprehension, computation, learning capacity, language, and the ability to differentiate.

**Figure 1:**
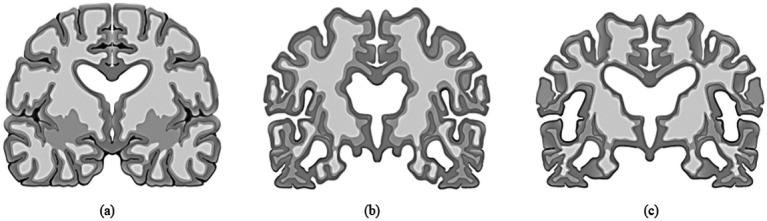
**(a)** Normal brain or control normal (CN), **(b)** Brain with mild cognitive impairment (MCI), **(c)** Alzheimer’s disease (AD) affected brain.

Most cases of dementia are either persistent or advance over time. This gradual progression may follow a course commencing with the cognitively normal (CN) stage, ultimately leading to the early cognitive impairment (MCI) stage and the late cognitive impairment (MCI) stage, and then on to Alzheimer’s disease (AD). Alzheimer’s disease is an irreversible form of neurodegeneration, so diagnosing it in its initial phases is critical before it manifests. The existence of the Apolipoprotein E4 (ApoE4) gene in an individual’s genome is one of the strongest predictors of Alzheimer’s disease risk. Researchers have been utilizing artificial intelligence (AI) methods such as deep Learning (DL) to handle complicated challenges in various industries, but medicine especially has been a focus of their attention. This trend is occurring concurrently with the fast rise of AI. Professionals in the field have significantly broadened the use of many deep learning models to discern and comprehend the distinct stages of Alzheimer’s disease. Recent studies using neuroimaging that make use of computer-assisted system research have made significant strides toward differentiating Alzheimer’s disease (AD) patients and cognitively normal patients. Although the binary categorization of individuals with AD and CN functioned beautifully, it is less effective than forecasting the initial stages shift from moderate cognitive impairment (MCI) to AD. The vast majority of studies ended in a binary category. As a result, they could not determine accurately whether the individual had MCI or the chance of them developing AD. Figure 1 illustrates the human brain affected by MCI, AD and CN.

Research studies use potential biomarkers to discover the disease, including fluid biological indicators, such as those found in cerebrospinal fluid (CSF), blood, saliva, urine, and tears. The dry biomarkers involving structural imaging, functional imaging, and visual indicators of Alzheimer’s disease have all been thoroughly investigated in various research, and the findings of these investigations have been analyzed and described to understand more about the disease and its stages (1). While specific research concentrates on clinical findings to explore the connection between progranulin levels in peripheral blood and the clinical manifestations of AD and MCI approaches, it also depends on computer aided diagnosis to obtain a higher accuracy in detection and prediction. The realm of artificial intelligence (AI) research has been used frequently, especially the Machine learning (ML) and deep learning (DL) models because they are considered to be two of the essential components of AI (2–4).

Machine learning is a systematic approach that generates automated and objective categorization, analyzes vast quantities of data that can be either complicated or simple, and differentiates between the minute changes which take place in brain imagery effectively (5). Pattern recognition methods that are based on machine learning are multidimensional and take into consideration certain inter-regional relationships that are indicative of various dispersed diseases. This information is used to assist in categorizing scans (6). Deep Learning has demonstrated promising potential in aiding clinical decision-making for several illnesses, including diabetes, retinal malignancy and Alzheimer’s Disease (7). The most significant advantage that deep learning has over more conventional, shallowly trained algorithms is its ability to acquire the most accurate predictive and classifying capabilities directly from unlabeled data (8). Deep Learning (DL) is a technique that analyzes data, discovers patterns, and processes that information analogous to how the neural networks in human brains do their tasks to solve complex decision-making issues (9–11).

Traditional techniques such as HOG and Harris corners offer robust, handcrafted descriptors that are particularly effective in capturing fine-grained spatial structures and texture features from MRI scans. These features are highly relevant in distinguishing between subtle anatomical differences associated with early stages of Mild Cognitive Impairment (MCI) and Alzheimer’s Disease (AD). Such handcrafted features are invariant to image transformations and can be critical in clinical datasets where variations due to scanner types or acquisition conditions exist. By using these traditional features as inputs to our classifiers—including a Deep Neural Network (DNN)—we provide the network with a rich, noise-resistant feature set, allowing it to focus more effectively on high-level pattern recognition without expending resources on learning low-level spatial gradients from scratch. This results in faster convergence, reduced overfitting, and improved generalization, particularly when the dataset size is limited. Moreover, our study shows that incorporating HOG and Harris corner-based descriptors before classification significantly improved accuracy (up to 95.6% with DNN), outperforming state-of-the-art deep learning models trained end-to-end. This suggests that the hybrid approach not only improves classification performance but also enhances model reliability, which is essential in critical applications such as medical diagnosis. The objective of this research endeavor was to construct an all-encompassing model for extracting features and performing multi-class classification of Alzheimer’s disease (AD) using machine the algorithms such as: Support Vector Machine (SVM), K-Nearest Neighbor (KNN), and a Deep Neural Network (DNN). It is challenging to define the different phases of Alzheimer’s disease due to the overlapping traits present in each stage. Extensive effort is required to classify this sickness into two or more stages. The objective of this research is to do a classification of the AD stages, which will consist of CN, MCI, and AD.

## 2 Background study

This section examines the contemporary ways to diagnose MCI and AD through the utilization of algorithms generated by computer-aided network models. Extracting features from images is a vital part of understanding the patterns. One such method is explained in the paper (12), the concept of Forstner’s and Harris’spoint-of-interest, which involves utilization of operators to determine the localized that demonstrates significant aberrations in both the temporal and spatial domains. The article has examined the use of linear separable filters for the purpose of identifying interest locations within the surrounding environment. The filters exhibit a response to intense movement and spatial–temporal corners. Furthermore, it has been proposed that the implementation of a voting framework based on the Hough transform be employed for the purpose of action recognition. This framework would employ spatiotemporal voting and make use of locally extracted X, Y, and T characteristics. The application of the human action recognition problem is investigated in this research, and several approaches, including transform-based descriptors and spatiotemporal interest points (STIPs), are offered in the article (13).

Medication and care are often the primary focuses of treatment modalities for Alzheimer’s disease (AD) that are now available. The early diagnosis may reduce the course of Alzheimer’s disease, hence postponing the emergence of full-blown dementia. In the absence of effective therapy for avoiding this critical disease, the detection at an early stage may slow the course of Alzheimer’s disease (14). By locating reliable illness-associated indicators, it may be possible to develop a credible early prognosis for Alzheimer’s disease (AD). In this respect, a wide variety of neuropsychological, biochemical, and genetic-based markers have been effectively used to monitor the course of dementia (15).

Machine learning (ML) in medical research may provide a productive way of navigating large amounts of information, essential for adequately identifying diseases. Machine learning, also known as the science of pattern learning, has the distinct advantage of handling large datasets, ultimately creating accurate prediction models (16). Machine learning makes the automated selection of significant predictors from an array of available inputs feasible. It is common practice to employ Magnetic Resonance Imaging (MRI) in conjunction with more complicated machine learning (ML) algorithms to differentiate between the brains of healthy individuals and those with mild dementia (17).

Over the last several years, various machine learning (ML)-based substantial research initiatives have been conducted to forecast dementia and Alzheimer’s disease (AD) and its exploitation in early detection. On the other hand, several of these previous findings relied on conventional machine learning classifiers, which do not need hyperparameter tuning or an ensembling approach. Because of this, the model’s accuracy and overall performance suffered as a consequence (18, 19). Deep learning algorithms, a different family of machine learning approaches, are doing very well in several areas, including voice recognition tasks, computer vision, natural language interpretation, and, most recently, medical data analysis. These models improve feature representation on MRI images using algorithms with a hierarchical structure and several layers (20).

The paper (21) suggests a CNN-based model using a patch-based classifier to identify AD with little computational expense and maximum improvement. Feature extraction and multi-operational processing on datasets have been made possible using deep learning-based algorithms. Models based on these techniques offer a higher capacity for feature representation on MRI images because of their hierarchical nature and many layers.

The research paper (22) employed a subset of RNNs called long short-term memory (LSTM) to predict sickness. To forecast the early stages of illness, this algorithm relies on patients’ historical data that is connected to their present activities and temporal data. This method employs three distinct layers—the cell layer, the post-fully connected layer, and the pre-fully connected layer—and bases its decisions on time series data. Instead of focusing on illness categorization, they discussed methods of prediction. Table 1 highlighted the employment of deep learning in the diagnosis and classification of Alzheimer’s disease.

**Table 1:** Deep learning in Alzheimers disease diagnosis and classification.

Paper	Dataset	Architecture	Classification	Highlights
(23)	ADNI	Two 3D CNN	AD, NC, MCI	The paper proposed a simple 3D convolutional neural network and exploits its model parameters to tailor the end to end architecture for the diagnosis of Alzheimer’s disease. The system made a simple and complex architecutre and trained model as two class and three class classification
(28)	ADNI	VoxCNN	AD, NC, EMCI, LMCI	Fast training CNN AD/LMCI/EMCI/Normal classification of 3D MRI images
(39)	ADNI	CNN-EL	AD vs. HC MCIc vs. HC MCI vs. MCInc	Phase 1 involved building three classifier ensembles based on axis slices. Phase 2 involved constructing a classifier ensemble based on output received from Phase 1.
(30)	ADNI	5LAyer CNN	CN-AD, AD-MC CN-MC AD-MCI-CN	The paper focused on the gray matter’s, to classify the AD and used 5 layer CNN model for the classification.
(31)	ADNI	CNN	AD vs. HC AD vs. MCI	The model has used denoising auto encoders to extract features from clinical and genetical data and 3D CNN for imaging data
(32)	ADNI	ADNet-DA	NC, MCI, AD	In this work, proposed an end-to-end deep 3D CNN for AD identification task, using the whole image volume as input. The methodology composed of three steps:-brain extraction and 3D CNN processing and domain adaptation
(36)	ADNI	3D CNN-SVM	AD vs. NC AD vs. MCI MCI vs. NC	The paper has modeled a 2D CNN,3D CNN, and 3D CNN-SVM architectures for binary classification of AD
(41)	OASIS	Deep Neural Network	AD vs. HC HC vs. Converted	The model has used deep neural network for prediction of AD and converted AD through EHR and MRI analysis

This research aimed to develop and verify a framework that can distinguish between individuals with AD and MCI using just structural MRIs of the brain. A powerful diagnostic marker has been developed to retrieve the characteristics from the MRI. Harris corner detection and the Histogram Oriented Gap (HOG) technique were used to extract features in the present.

Limitations of earlier research include the absence of an age factor and the use of imaging data collected from a single location, both of which reduce the generalizability of results, as age plays a vital role in extending the life expectancy of the patients. In light of this, our study’s primary goal and novelty was to diagnose and classify AD from MCI and CN for early onset individuals.

The paper’s structure is organized in the following way. The article starts with an introductory section, providing a contextual overview, a comprehensive background study, and an examination of pertinent contributions to the proposed research. The proposed technique is then elaborately described in Section 3. Furthermore, Section 4 elucidates the categorization strategies utilized in the conducted tests. Subsequently, Section 5 provides an exposition of the evaluation metrics employed in the study. Following this, Section 6 gives the findings and their corresponding analysis. Lastly, the article culminates with a comprehensive examination of critical remarks in Section 7.

## 3 Methodology

**Figure 2:**
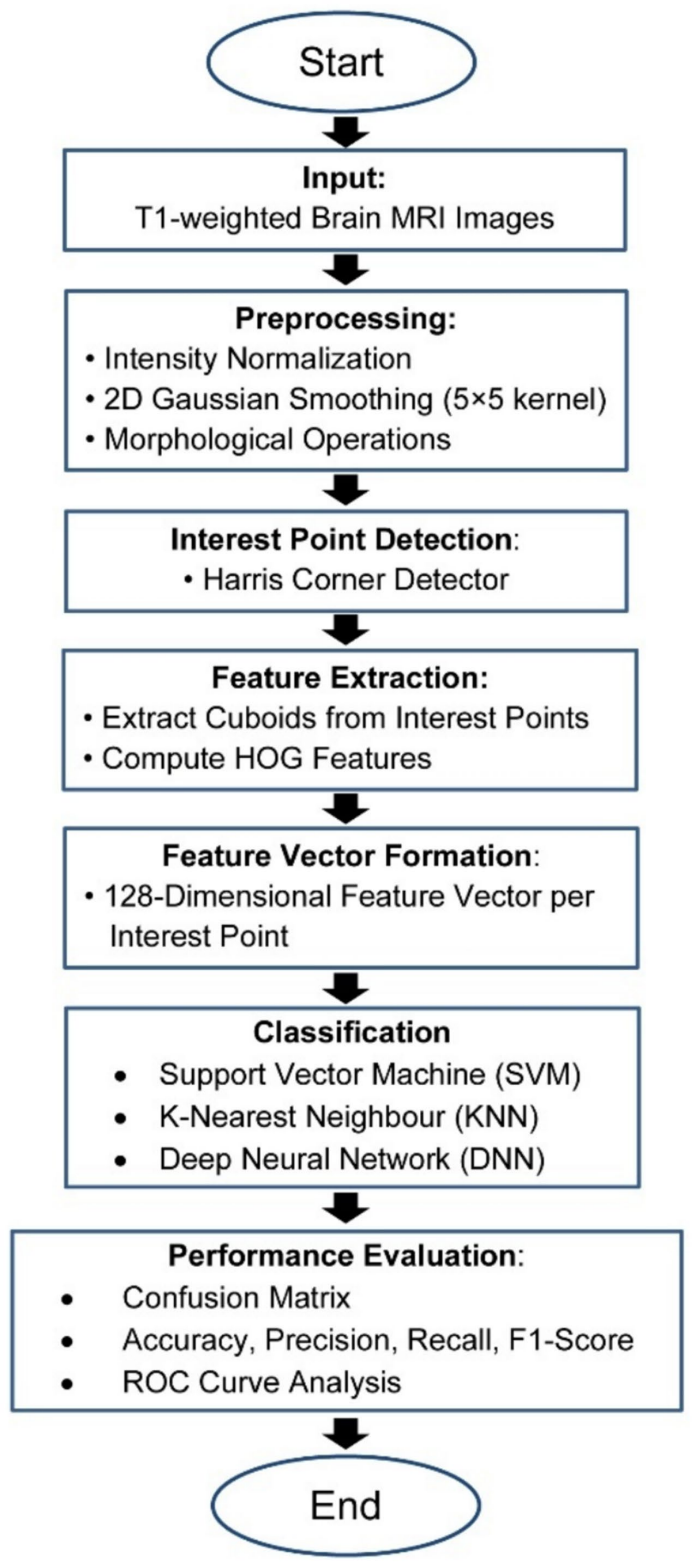
Flowchart of the MRI-based Alzheimer’s classification pipeline.

The present study has devised a comprehensive structure for categorizing the stages of AD using MRI data, encompassing a spectrum that spans from those without dementia to those with severe AD. The fundamental objective of the proposed strategy is to decrease reliance on extensive datasets and achieve superior performance in identifying the stages of Alzheimer’s disease (AD) compared to classification methods. The Flowchart of the MRI-based Alzheimer’s classification pipeline from preprocessing to feature extraction and classification is show in the Figure 2.

**Figure 3:**
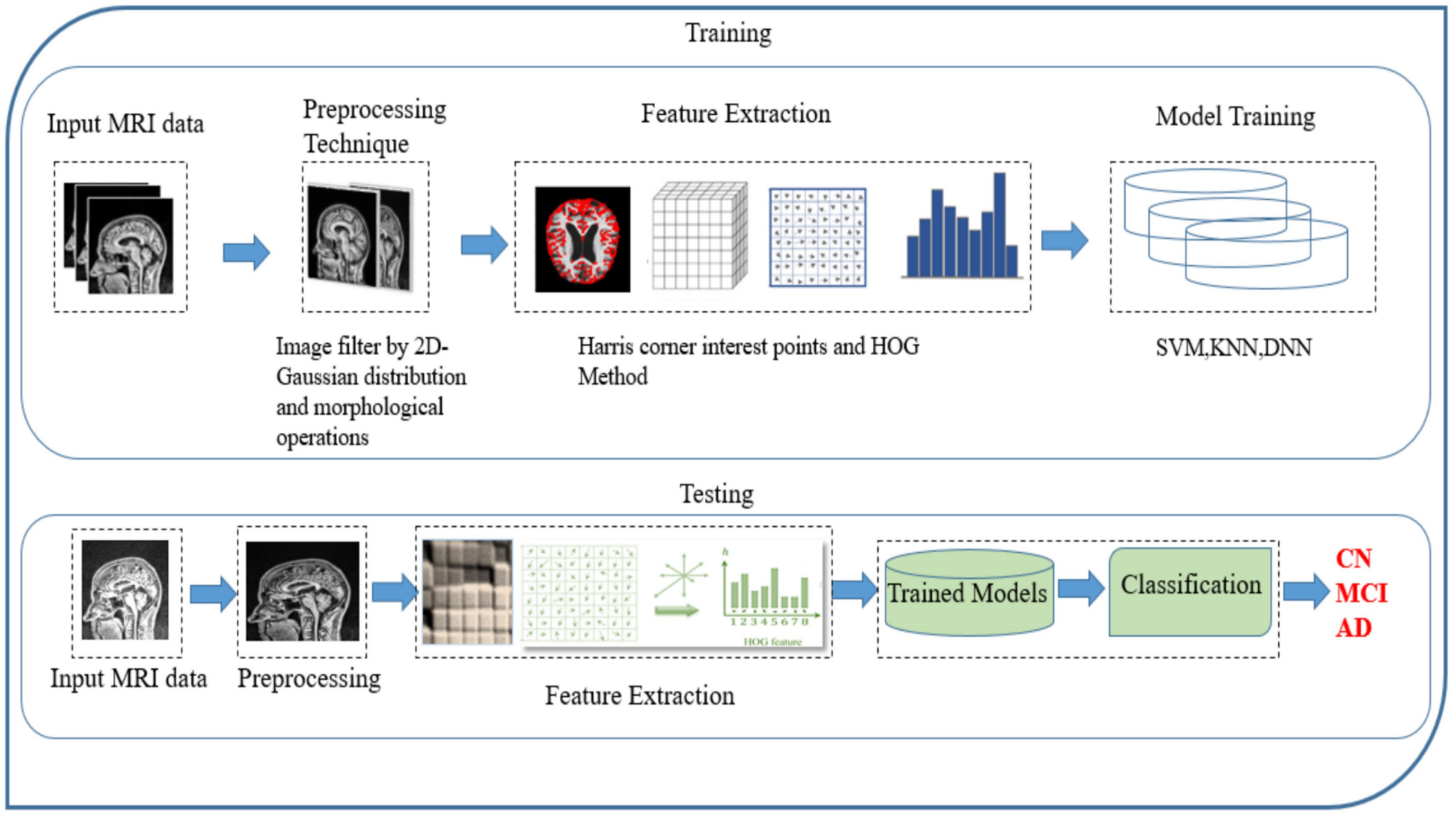
Framework model for Alzheimer’s disease classification.

The MRI data used in this study were obtained from the Alzheimer’s Disease Neuroimaging Initiative (ADNI) database. The ADNI dataset, a multicenter, longitudinal resource, provides structural MRI scans, alongside clinical, demographic, and biomarker data for subjects diagnosed with AD, MCI, and cognitively normal (CN) controls. Data are collected at multiple time points to enable analysis of disease progression. The Alzheimer’s Disease Neuroimaging Initiative (ADNI) dataset is an extensive, multicenter, longitudinal benchmark designed to facilitate the early detection and monitoring of Alzheimer’s Disease (AD) and Mild Cognitive Impairment (MCI). It provides high-resolution, quality-controlled structural MRI and PET scans acquired according to standardized protocols across multiple clinical sites. Demographic entries such as age, sex, diagnostic category (AD, MCI, or cognitively normal controls), and longitudinal visit labels are included, enabling analysis of disease progression and conversion. In addition to imaging, ADNI offers rich clinical annotations, neuropsychological scores, genetic information (e.g., APOE genotype status), and relevant biochemical markers. All MRI data undergo protocol harmonization and quality assessment before public release via the Laboratory of Neuroimaging (LONI) at USC, ensuring reliability and reproducibility for AI and neuroimaging research. This comprehensive dataset underpins state-of-the-art machine learning algorithms, supporting robust model training, validation, and benchmarking in the context of dementia classification and progression studies. The dataset primarily includes individuals aged 55 to 90 years, with a demographic distribution heavily skewed toward Caucasian participants from North America. While ADNI provides high-quality imaging and diagnostic labels, the limited ethnic and age diversity may introduce potential biases, affecting the model’s generalizability to more diverse global populations. Future work should focus on validating the proposed method on datasets that are more representative of different age groups, ethnicities, and imaging protocols. All brain MRI scans used in this study were in the NIFTI format (.nii or .nii.gz), which is standard in neuroimaging due to its support for multidimensional, volumetric data and rich metadata. NIFTI files facilitate precise spatial referencing and compatibility with medical imaging toolkits. All MRI volumes were resampled to a uniform spatial resolution (voxel size) to ensure consistency across the dataset. This step aligns the physical dimensions of each scan, necessary for reliable downstream analysis. A Gaussian convolution approach is employed to smooth all pictures to achieve effective feature extraction and classification. The smoothing process uses a matrix size of 5 × 5. All MRIs were registered to a common anatomical space (typically MNI or similar atlas) via affine or nonlinear registration. This enables voxel-to-voxel correspondence, crucial for group comparisons and machine learning. Non-brain tissues (e.g., skull, scalp) were removed from the MRIs using automated brain extraction tools (SPM). This isolates brain tissue, reducing irrelevant data and enhancing focus on brain morphology. After all MRI scans underwent standard preprocessing (normalization, and skull stripping), various data augmentation methods were applied to further expand the effective training set and reduce overfitting. Augmentation techniques included random rotation, flipping, shifting, scaling, cropping of brain ROIs, and injection of Gaussian noise. These augmentations increased data variability, preventing the model from memorizing specific examples and improving its generalization to unseen data. The suggested methodology presents a substantial enhancement and contribution to the field of detection and classification. The description of the suggested technique is shown in Figure 3. The following is a list of the key contributions that this paper makes: (i) The proposed methodology is to identify the early stages of MCI and AD among the younger onset of ages 45 to 60 (ii) A 2D Gaussian approach has utilized to enhance the image quality by removing the noise and smoothening the image. (iii) Processed images under goes for feature extraction using Harris corner detection and Histogram oriented gradients (HOG). iv) Finally, the Classification is achieved with SVM, KNN, and DNN classifiers and the performance is analyzed.

### 3.1 Data preprocessing and augmentation

**Figure 4:**
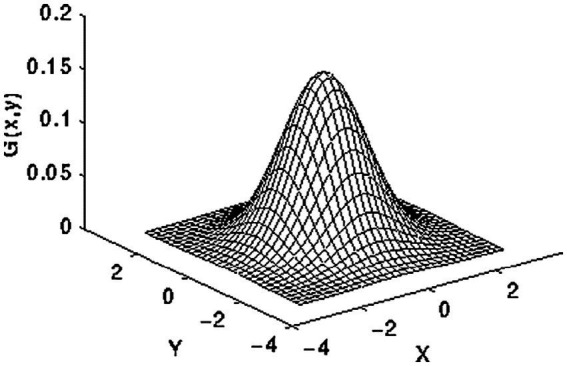
2-D Gaussian distribution.

Intensity normalization is a commonly utilized approach in the domain of image processing. It entails altering the distribution of intensity values of pixels, often known as histogram stretching. The methodology’s goal is to modify the input image in order to connect it more effectively with natural or recognizable impressions (23, 24). Initially, the 2D Gaussian smoothing operator is employed for image smoothing, which involves the reduction of detail and noise. In this context, it exhibits similarities to the mean filter, utilizing a 5 × 5 kernel for its operation. This kernel has some unique properties. The 2D Gaussian applies the following formula in Equation 1 for the elevation process of the image, and the distribution depicted in Figure 4 exhibits the standard deviation, with a value of 1, along the x-axis and y-axis.


(1) $$G\left(x,y\right)=\frac{1}{2\Pi \sigma^{2}}e^{-\frac{\left(x^{2}+y^{2}\right)}{\left(2\sigma^{2}\right)}}$$

**Figure 5:**
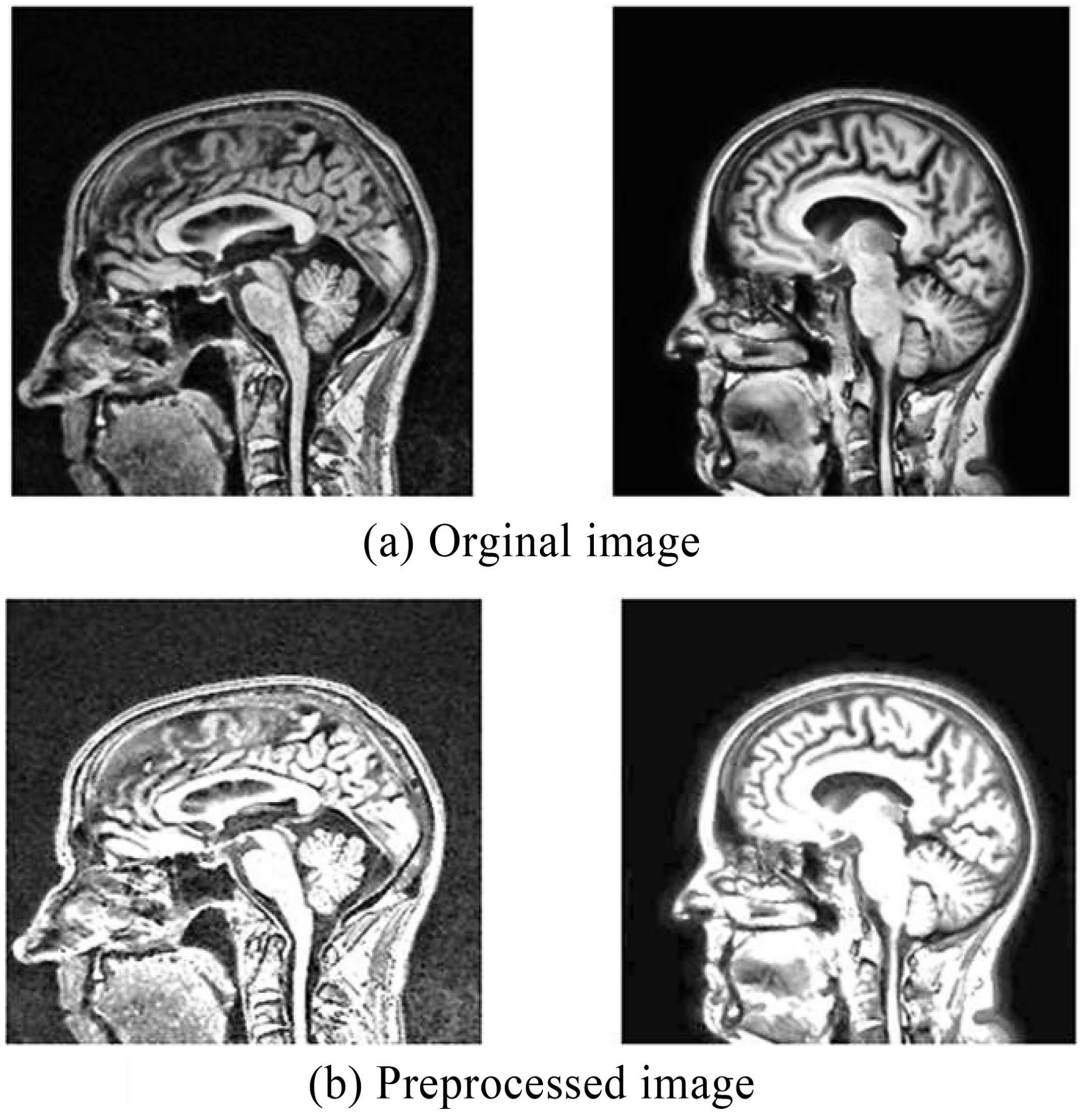
**(a)** Original MRI image before preprocessing. **(b)** Preprocessed MRI image.

Finally, applied morphological operations to remove the small objects and thin lines from an image while preserving the shape and size of larger objects in the image. Preprocessed brain MRI is depicted in Figure 5.

Data augmentation techniques, such as random rotation, flipping, shifting, scaling, cropping, and noise injection, were employed during the training phase. These methods artificially enhance the diversity of the training set by generating varied versions of the original MRI scans. Increasing data diversity in this way exposes the model to a wider range of plausible scenarios and anatomical variability, which prevents it from memorizing training examples and thus reduces the risk of overfitting. As a result, the model learns more robust and generalizable features, improving its performance on unseen patient scans.

### 3.2 Interest point identification

The present research utilizes the Harris interest point detector owing to the high degree of invariance it possesses with regard to noise, illumination scaling and. An image’s spatial and temporal dimensions are used to confine the locations of interest points within the image frames. The Harris corner detector leverages the local autocorrelation function as its primary analytical foundation. Around the corner, the visual intensity will shift significantly in several distinct manners (25). When the shift [u, v] is applied to the image, the algorithm calculates the intensity change as follows in Equation 2:


(2) $$E\left(u,v\right)=\sum_{x,y}w\left(x,y\right)\;{\left(\left(I(x+u,y+v\right)-I\left(x,y\right)\right)}^{2}$$

Where w(x,y) is the rectangular window function, it give weight to the underneath pixels. The function (I(x+u,y+v) and I(x,y) is the shifted and original intensity values. Detecting corners with a maximum range of intensity differences is necessary In consideration of this, a Taylor expansion is used to generate an approximation of the shifted image, and a score is ultimately computed to identify relevant points, as shown in the following Equation 3:


(3) $$R=\det\left(M\right)-k{\left(trace\left(M\right)\right)}^{2}$$

**Figure 6:**
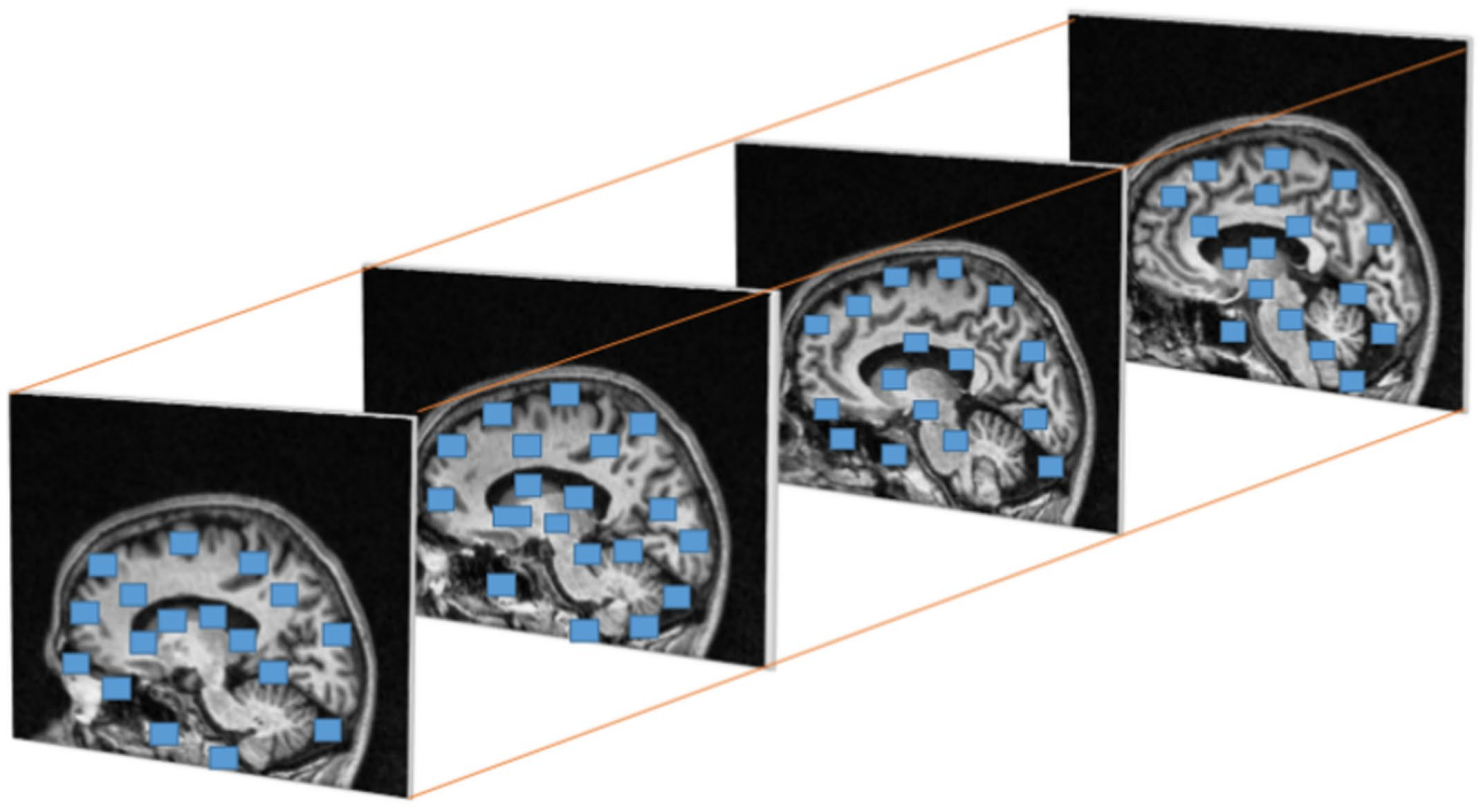
Cuboids from Harris corner interest points in brain MRI.

Where det(M) is $\lambda_{1}\lambda_{2}$ , trace(M) is $\lambda_{1}+\lambda_{2}$ and the $\lambda_{1}\;and\;\lambda_{2}$ are the Eigen values of M. When a window with value R exceeds the threshold point is defined as interest points with corner. Figure 6 represents the discovered interest points within the MRI slices of the dataset. Points of interest in the local maximum response function are spatiotemporal points that have been highlighted.

### 3.3 Feature extraction

Histogram Oriented Gradients (HOG) features were frequently utilized (and continue to be used) in a variety of computer vision due to its excellent analytical power, endurance to changes in illumination, clarity, and ease of deployment. Here, we demonstrates a methodology for extracting the variants of HOG features from the cuboids with the highest interest points; this variant exists in a spatiotemporal domain and has sufficient discriminative capacity to define and measure the textures in brain MRI scans accurately. To accomplish this goal, two fundamental units of computation the cell and the block must first be locally specified. The block size for each Histogram of Oriented Gradients (HOG) feature is defined in terms of cells, where each cell is characterized by pixel’s size (26).

The extraction of HOG features involves the initial computation of gradient and direction values for every pixel point (x, y). To achieve this, we first determine the gradients of the image as and in the x and y directions using Equations 4, 5.


(4) $$g_{x}=I\left(r,c+1\right)-I\left(r,c-1\right)$$

(5) $$g_{y}=I\left(r-1,c\right)-I\left(r+1,c\right)$$

where´ r’ and´ c’ are the respective rows and coloumns. Now calculate each image’s magnitude and angle using the Equation 6:

(6) $$\mathrm{Magnitude}\;\left(\mu \right)=\sqrt{g_{x}^{2}+g_{y}^{2}}\;Angle\;\left(\theta \right)=\mathrm{atan}\left(\frac{g_{x}}{g_{y}}\right)$$

After obtaining each pixel’s gradients, its matrices must be calculated. The gradient magnitude and angular orientation of each pixel in the cells are converted into a set of angular bins. For every image undergone with pixel orientation, a histogram is built for each cell and its weight is computed using Equation 7:


(7) $$\delta =b+0.5-\frac{g_{y}}{\pi \;}m$$

Where m represents the histogram bin an element belongs to, and b is the set of bins in the histogram. The present research ensured updating the values of two neighboring bins to eliminate aliasing. The procedure is outlined in Equation 8:


(8) $$\tilde{\delta }=(1-g_{x}),\overset{\vee }{\delta }=g_{x}g_{y}$$

**Figure 7:**
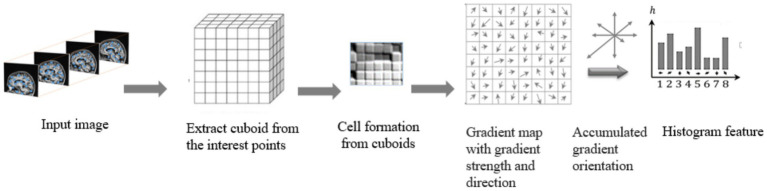
Extraction of raw features with HOG method.

The weighted ballots are compiled into histogram sections across the local spatial regions, known as cells. Extraction of HOG features from a brain MRI input image is shown in Figure 7.

The present research utilizes an adaptive approach to enhance the depiction of Histogram of Oriented Gradient (HOG) characteristics, in which the degree of similarity between image fragments is used to depict the spatial relationship among local regions (27). To accomplish this, the orientation bins of the 8-bin HOG histogram generated from an individual cell are progressively altered by a value (=0, 1, 2, 7) to yield a total of 8-bin histograms. Next, the transformed Histogram of Oriented Gradients (HOG) feature characteristics of two cell areas, denoted as c1 and c2, are explicitly calculated by evaluating the dimension correlations of the orientation-shifted 8-bin histograms using Equation 9:


(9) $$b_{c1,c2}\left(k,\varepsilon \right)=\left\{\begin{array}{c} 1\\ 0 \end{array}\;\begin{array}{c} ,if\;v_{c1\left(k\right)\geq }\;v_{c2\left(\left(k+\varepsilon \right)\%8\right))}\\ ,otherwise\; \end{array}\right\}$$

The utilization of binarized orientation-shifted histograms in the extraction of HOG features offers the possibility of obtaining a more concise and resilient representation of these characteristics. Additionally it lowers the computation time of the overall process. This, in turn, invariably contributes significantly to both increased speed and improved accuracy in object recognition. The following algorithms explain Feature extraction from the cuboids obtained through interest points.

1 function HOG (image, cell_size, block_size, num_bins):
2 Compute gradients of image as I_x_ and I_y_ in x and y directions.
3 Calculate the gradient magnitude and angle.

magnitude = compute_magnitude(I_x_, I_y_).

angle = compute_orientation(I_x_, I_y_).

4 Divide the cuboid into cells.

cells = divide_cells(magnitude, angle, cellsize).

5 Calculate the histogram for each cell obtained from the cuboids.

Hist = [].

For cells in cells:

Hist = Calculate_Hist(cell, number _of_bins).

Histogram.append (Hist).

6 Combine the calculated histograms into blocks.

Descriptor_block = [].

For i in range (number_of_cells_x- blocksize+1):

For j in range (number_of_cells_y, blocksize+1):

Blocks = Hist [i: i + blocksize, J: J + blocksize].

Descriptor_block = normalize_descriptor_block (block_hist).

Block_descriptor.append (Descriptor_block).

7 Concatenate the descriptors to obtain the resultant HOG descriptor.

HOG_descriptor = Concatenate (Descriptor_blocks).

8 Return the HOG descriptor of the image.

Return_HOG_descriptor.

The size of the cells, blocks and the number of bins are the set of parameters that need to be identified before the function ‘HOG’ call. The cell size represents the size of the cells required to compute the descriptor. The HOG histogram’s block size is set by the blocksize parameter, and the number of bins is set by the number_of_bins parameter. Calculate the Gradient around each interest point’s 16 × 16 pixel area. In order to create a feature vector with 128 dimensions (4 × 4 × 8), the region is first separated into 4 × 4 sub-regions. For each sub-region, the 8-bin gradient orientation h (k), with k = 0 to 7, is computed. Finally, creating a feature vector with a size of 128 dimensions by combining every gradient orientation histogram in the interest point’s area.

#### 3.3.1 Parameter selection and sensitivity analysis

The choice of key parameters in this study was guided by a combination of prior research findings and preliminary experimental validation to optimize classification accuracy and model generalization.

- HOG Cell and Block Size: A cell size of 8 × 8 pixels and block size of 2 × 2 cells were selected based on standard practices in medical imaging, which balance local texture capture and computational efficiency. Larger cells tended to smooth over fine anatomical details critical for early-stage Alzheimer’s detection, while smaller cells increased noise sensitivity without substantial performance gain.
- SVM Kernel Type: The Radial Basis Function (RBF) kernel was chosen after empirical comparison with linear and polynomial kernels. The RBF kernel demonstrated superior capability to handle the non-linear decision boundaries inherent in MRI feature distributions, resulting in a 3–5% higher classification accuracy compared to linear kernels.
- DNN Hyperparameters: The number of hidden layers (9 layers), neurons (256 in the input layer), activation function (ReLU), optimizer (Adam), learning rate (0.01), and batch size (24) were selected through iterative grid search experiments. We observed that increasing the number of hidden layers beyond 9 led to overfitting, while lower layers resulted in underfitting, confirming 9 layers as the optimal depth for this dataset.

Sensitivity experiments were conducted to validate the robustness of parameter choices, as summarized in Table 2:

**Table 2:** Sensitivity analysis of key parameters on classification accuracy.

Parameter	Variation tested	Accuracy impact
HOG Cell Size	4 × 4, 8 × 8, 16 × 16	8 × 8 yielded highest accuracy (+4%)
SVM Kernel	Linear, Polynomial, RBF	RBF outperformed others by ~3%
DNN Hidden Layers	6, 9, 12	9 layers achieved best results
Learning Rate	0.001, 0.01, 0.1	0.01 provided faster convergence

The sensitivity results confirm that the selected parameters yield a balanced trade-off between model complexity, training time, and classification accuracy.

## 4 Classification methods

In this work, the effectiveness of the classifier in the ADNI dataset is evaluated using. The following classifiers were utilized in this study:

### 4.1 SVM

**Figure 8:**
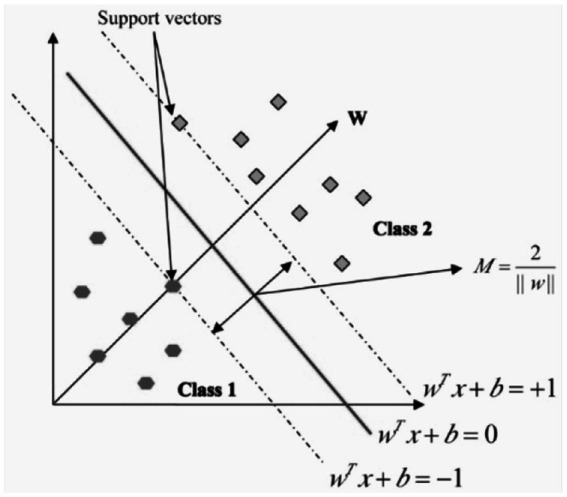
Hyperplane in linear support vector machine.

In the field of pattern recognition, the Support Vector Machine, or SVM, is a method that is frequently utilized for the classification of images. Utilizing essential pattern recognition, it is able to realize higher levels of success in the application of optimization theory, and the use of the kernel learning algorithm is the primary focus of this method. With the assistance of constructing a model, SVM attempts to make predictions about the target values based on the testing set (28). In binary classification, the hyper plane is represented by the equation wx + b = 0, where w ∈ $R^{n}$ and b ∈ R are engaged in the process of separating two classes in the separate space Z. the maximum margin is assumed to be $M=\frac{2}{|\left|w\right||}$ and it is shown in the Figure 8.

The incorporation of non-negative slack variables *β* is employed to optimize the margin size and mitigate the occurrence of learning and training errors. Optimizing Equations 10, 11 will result in the acquisition of the soft margin classifier.


(10) $$\underset{w,b,\beta }{\min}.5w^{T}w+C\overset{l}{\underset{i=1}{\sum }}b_{i}$$

(11) $$y_{i}(w^{t}\varphi \left(x_{i}\right)+b\geq 1-\beta_{i,}\beta_{i,}\geq 0$$

When a linear separation of the training data is not possible, a kernel function is used to turn the input space into a high-dimensional space. Table 3 outlines a few of the most important features that are associated with kernel functions.

**Table 3:** SVM Kernel types.

Types of kernel functions
Linear: $\;G\left(x,x_{n}\right)=sum\left(x.x_{n}\right),where\;x,x_{n}\;are\;thedataforclassification$ Polynomial P: Gx,xn=x.xn^d,whereGx,xnrepresentsthedecisionboundary,ddenotesthedegree Radial base function: $\begin{array}{l} G\left(x,x_{n}\right)=\exp(-gamma\left(\mathrm{||}\left|x-x_{n}\right|\mathrm{||}{)}^{2}\right),\;wherethevalueof\\ gammavariesfrom\;0\;to\;1 \end{array}$ Sigmoid: $G\left(x,z\right)=\tanh\left(\;\alpha x^{t}z+c\right)$

The Multiclass Support Vector Machine is helpful in the building of an N-binary classifier because it separates a particular class from the other classes. The training sets for the ith class primarily comprise positive labels, whereas the other labels are predominantly associated with negative connotations. The support vector machine corresponding to the i-th decision function is responsible for decrypting it.

### 4.2 K-nearest neighbor

The K-nearest neighbors (KNN) algorithm is a classification method that relies on the proximity of neighboring data points to create predictions, as opposed to the traditional approach of developing a predictive model. The calculation of this method relies on the concept of nearest neighbors. The K-Nearest Neighbors (KNN) prediction analysis approach is employed to identify the k-nearest neighbors of data samples, with the purpose of making predictions. The Euclidean function is used to compute the distance between two points, which also assists in determining whether or not the two points have any similarities. The collection of labeled data samples is denoted by the notation C = (x1, x2, etc.). A given data set is denoted by this notation. The nearest neighbor classifier gives test point ‘D’ the label that is linked with its neighbor in C that is the closest. The point D is put into the appropriate category using the K- nearest neighbor classifier, which does this by associating it with the label that is seen most often among the samples that are closest to it (29). In this iteration of the method, the most important parameter is X. The Euclidean distance is employed to compute the distance between training and testing data points as $\;x,x_{i}$ . $d_{E}=\left(x,x_{i}\right)=\left(\overset{n}{\underset{i=0}{\sum }}\mathrm{||}x-x_{i}\mathrm{||}\right)$ to determine how far apart the two sets of points.

### 4.3 Deep neural network

A Deep Neural Network (DNN) is characterized by its multi-layered structure, where interconnected neurons form the network. This gives it the ability to model intricate patterns and abstractions. It belongs to a large family of machine learning models that are collectively referred to as deep learning. This kind of knowledge acquisition seeks to tackle various problems by modeling the framework and operations of the human brain. When using a DNN, data is sent feedforward across the network, where it travels via numerous layers of neurons. Each layer is made up of a collection of neurons or nodes and the weights that are assigned to the links that are made within these neurons. The learning course, also known as training, involves modifying these weights so that the neural network can learn to approximate the intended output given a specific input (30). Before beginning the training process, some hyperparameters must be configured for deep neural networks. These hyperparameters substantially influence the overall efficiency of the network and its capacity to converge quickly and generalize information.

The following are some of the fundamental components that make up a DNN:

The initial surface of the network, the input layer, is where data are introduced into the modeling process.

Hidden Layers are the layers that are positioned in between the input and output layers of a structure. These layers are in charge of discovering and accurately portraying intricate patterns and characteristics in the data they are given.

**Figure 9:**
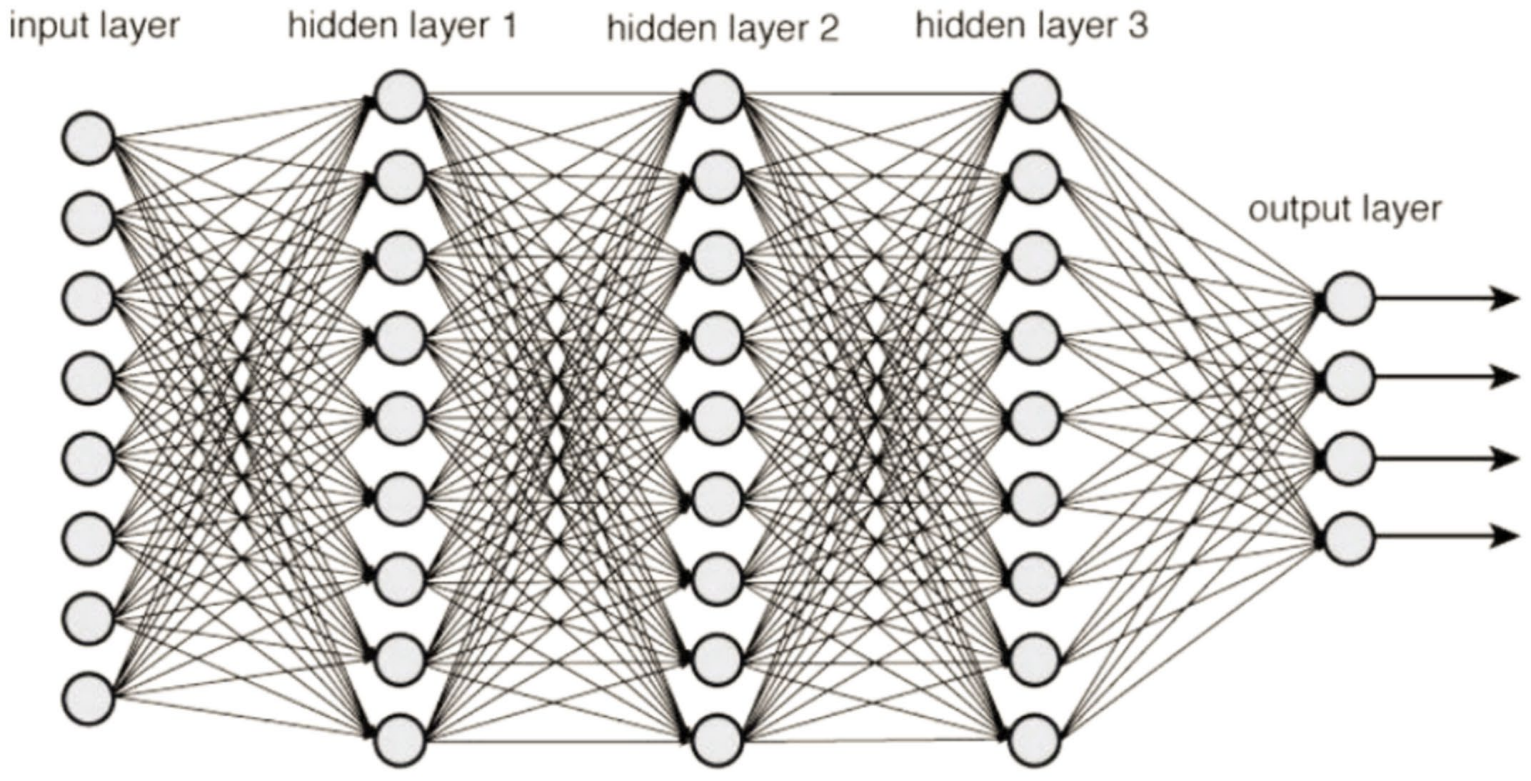
DNN framework.

The last layer of the network, the output layer, is responsible for producing the output that has been anticipated based on the representations that have been learnt in the hidden layers. Figure 9 illustrates a sample explanation of deep neural networks.

It is essential to tweak these hyperparameters depending on the particular dataset and the situation at hand. Experimentation is a common part of this process, which may be carried out using strategies such as grid search and random search, as well as more complex approaches such as Bayesian optimization and evolutionary algorithms. The objective is to identify the hyperparameter settings that, when combined, will produce a deep neural network that is both high-performing and generalizable. Before beginning the training process, there are a number of hyperparameters that must be configured for deep neural networks. These hyperparameters have a substantial influence on the overall efficiency of the network as well as its capacity to converge quickly and generalize information. The following are some significant hyperparameters:

Number of Hidden Layers: One of the most important hyperparameters to consider is the number of hidden or concealed or layers in the network. Deeper neural networks can learn more intricate representations,

Number of Nodes in Each Hidden Layer: The ability of the model to learn complicated patterns is also impacted by the overall number of nodes included inside each hidden layer. Adding additional nodes to the network model will often result in a surge in its capacity;

Activation Functions: Activation functions enable the network to simulate complicated interactions by introducing non-linearity into the system and giving it the ability to model connections between nodes.

Learning Rate: During gradient descent optimization, the size of each step is determined by the learning rate, which is a percentage. A greater learning rate may save the amount of time needed for training, On the other hand, having a poor learning rate might cause convergence to take longer or even cause one to get mired in a local minimum.

Batch Size: When talking about training a model, “batch size” refers to the amount of samples that undergo processing before the weights of the model are updated.

Number of Epochs: The total amount of time that the complete dataset is run through the network while it is being trained is represented by the number of epochs.

The optimizer is responsible for deciding which approach will be used to update the model’s weights while being trained. Gradient Descent, Adam, RMS-prop, and a number of other optimizers are among the most common.

It is essential to tweak these hyperparameters depending on the particular dataset and the situation at hand. Experimentation is a standard part of this process, which may be carried out using strategies such as grid search and random search, as well as more complex approaches such as Bayesian optimization and evolutionary algorithms. The objective is to identify the hyperparameter settings that, when combined, will produce a deep neural network that is both high-performing and generalizable (31, 32).

## 5 Evaluation metrics

Metrics of performance are used in the process of determining how successful the model or algorithm. These metrics convey quantifiable measurements of how well the model is working on a certain job, such as classification, regression, clustering, or other sorts of learning tasks. To ensure reproducibility and robustness, a 5-fold cross-validation strategy was adopted throughout all experiments. The dataset was randomly divided into five equal subsets. In each iteration, four subsets (80%) were used for training and internal validation (with an 80:20 split between training and validation data), while the remaining subset (20%) was reserved for testing. This process was repeated across five folds, ensuring that every sample appeared once in the test set. The final classification performance for each model (SVM, KNN (37), and DNN (38, 40)) was reported as the average of the results obtained across all folds. This approach reduces variability and provides a more reliable estimate of the model’s generalization ability. The confusion matrix for the image classification has true positive (TP), false positive (FP), true negative (TN), and false negative (FN) class values. It is considered a “success” when the classifier correctly forecasts the outcome of class at each occurrence; otherwise, it is considered as an “error.” The error rate, which is a fraction of the mistakes produced throughout the whole collection of examples, is used to provide a broader assessment of the efficiency of the classifier (42–45). Table 4 describes the Confusion matrix for classification.

**Table 4:** Sample confusion matrix for classification.

		Actual values	
		Positive	Negative
Predicted values	Positive	True Positive (TP)	False positive (FP)
	Negative	True Negative (TN)	False Negative (FN)

Accuracy may be defined as the ratio of properly categorized examples to the entire number of instances within a specific dataset.


$$\text{Accuracy = }\frac{TN+TP}{TN+TP+FP+FN}$$

Recall represents the percentage of genuine positive predictions out of all real positive occurrences in the dataset. This metric is also known as sensitivity.


$$\text{Recall = }\frac{TP}{TP+FN}$$

The F1 score is the harmonic mean of the recall and accuracy scores. It offers a balance between accuracy and recall, which is particularly helpful when there is an imbalance in the class distribution.


$$\text{F1-score = }\frac{2TP}{2TP+FP+FN}$$

Specificity, also known as the True Negative Rate, assesses the percentage of accurate negative predictions made in comparison to the total number of real negative occurrences.


$$\text{Precision = }\frac{TP}{TP+FP}$$

## 6 Results and discussions

Extensive research has been conducted on the automated categorization of disease’s stages, namely control normal (CN), Mild Cognitive Impairment (MCI), and Alzheimer’s Disease (AD). The collected MRI has undergone preprocessing with 2D-Gaussian distribution and morphological operations. Using the Harris detector, the initial step is to locate every point of interest in the image being examined. This operator relies on the local image structure described by the auto-correlation matrix. The approach presented in this study utilizes a combination of the Harris corner and Histogram of Oriented Gradients (HOG) methods for feature extraction. Additionally, trained models like Support Vector Machines (SVM), k-Nearest Neighbors (KNN), and Deep Neural Networks (DNN) are applied for the purpose of classifying different illness stages. The SVM algorithm is widely employed in classifying datasets that are binary or multiclass in nature. The utilization of a kernel function to map data to a high-dimensional space in a non-linear manner results in the generation of an optimum segmentation plane for segregating individuals. The use of Harris corner detection and HOG for handcrafted feature extraction substantially reduced the dimensionality and complexity of input data, which in turn decreased the training time for the classifiers. Unlike full 3D CNN architectures that operate directly on volumetric MRI scans and require extensive computational resources, our approach offloads feature representation to a fast and efficient extraction step. This led to a reduction in training time from several hours to approximately 4–5 min per volume on an RTX 3060 GPU, making the method practical for clinical applications.

**Figure 10:**
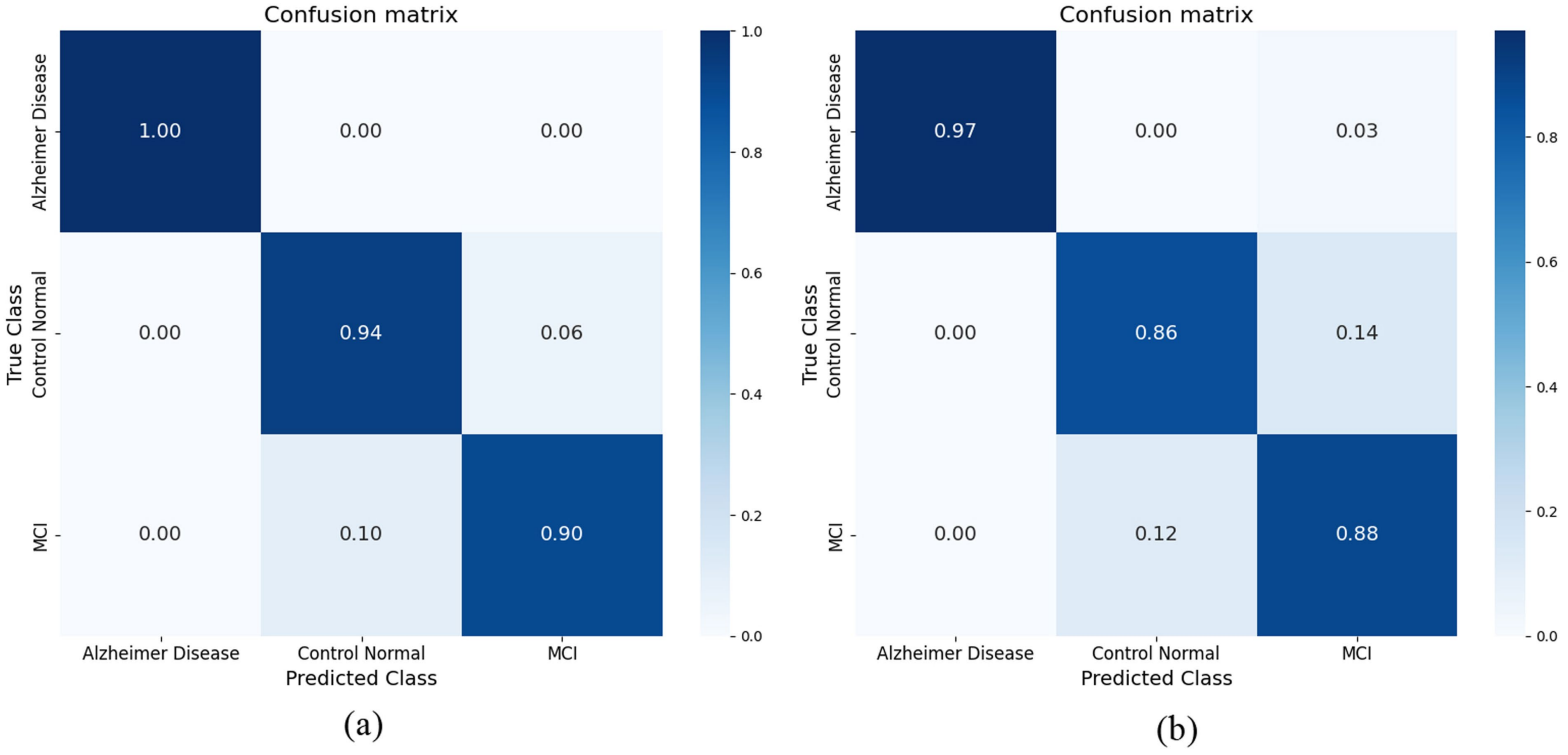
**(a)** Confusion matrix achieved for SVM model. **(b)** Confusion matrix achieved for KNN model.

Our hybrid approach, combining ResNet-18 with attention layers and HOG-based features, yielded unexpectedly strong improvements, especially in distinguishing MCI from cognitively normal subjects—a notably challenging classification. The model showed robustness to class imbalance and maintained stable generalization on unseen validation data. Attention mechanisms enhanced interpretability by highlighting relevant brain regions linked to Alzheimer’s pathology. ResNet-18 was selected for its balance of depth and computational efficiency, with attention layers aiding feature discrimination and noise reduction. The research study employed the radial basis function (RBF) kernel. Figure 10a illustrates the confusion matrix of the Support Vector Machine (SVM) classifier, demonstrating a perfect correspondence between the predicted and actual values. The Support Vector Machine (SVM) model using the Radial Basis Function (RBF) kernel has achieved an average prediction accuracy rate of 91.5%. The confusion matrix obtained for the Support Vector Machine (SVM) classifier is illustrated in Figure 10a. The performance of the Model in accurately classifying Alzheimer’s Disease (AD) from Mild Cognitive Impairment (MCI) and Cognitively Normal (CN) individuals is commendable. However, it exhibits a tiny degree of uncertainty when distinguishing between the MCI and CN groups. The K-Nearest Neighbor (KNN) technique is employed as the second machine learning classifier in the present study. This technique sets the value of k to 1. Figure 10b illustrates the confusion matrix that has been produced using the K-nearest neighbors (KNN) classifier. When attempting to categorize AD from CN and those with MCI, the classifier successfully matched the real value and predicted value to a higher degree of accuracy. However, it encountered difficulties in accurately identifying individuals with MCI and CN. The K-nearest neighbors (KNN) algorithm achieved an average accuracy rate of 88%.

The development of the deep neural network (DNN) model encompassed the establishment of several neural network parameters, including the specification of 256 nodes for input and 3 nodes for output, 10 hidden layers were utilized, along with the Adam optimizer. The hyper tuned DNN classifier was able to classify and obtained a result of 96%. The below table explains the hyper tuned DNN model. The model has used nine hidden layers to get the optimum results. When the count of hidden layers is incremented, the model’s performance starts to decline and results in low performance. The current development shows that the network model consistently achieved satisfactory results with nine hidden layers. Table 5 shows the hyper-tuned DNN network parameters. To mitigate overfitting in the deep neural network (DNN) model, several regularization techniques were employed. Dropout layers with a dropout rate of 0.3 were inserted between hidden layers to randomly deactivate neurons during training, thus preventing the model from becoming overly dependent on specific nodes. Additionally, L2 regularization (weight decay) was applied to penalize large weights and promote simpler models, with a regularization coefficient set to 0.001. Early stopping was also implemented, monitoring the validation loss during training and halting the process if the loss did not improve for 10 consecutive epochs, thus avoiding over-training. Data augmentation was not performed, as the input MRI data had already undergone preprocessing and normalization to enhance consistency. These techniques collectively contributed to improving the model’s generalization capability while maintaining high classification accuracy across folds.

**Table 5:** DNN hyper tuned parameters.

Parameters	Ranges
Hidden layers	9 layers
Optimizer	Adam
Activation function	Relu
Number of epochs	50
Learning rate	0.01
Batch Size	32

**Figure 11:**
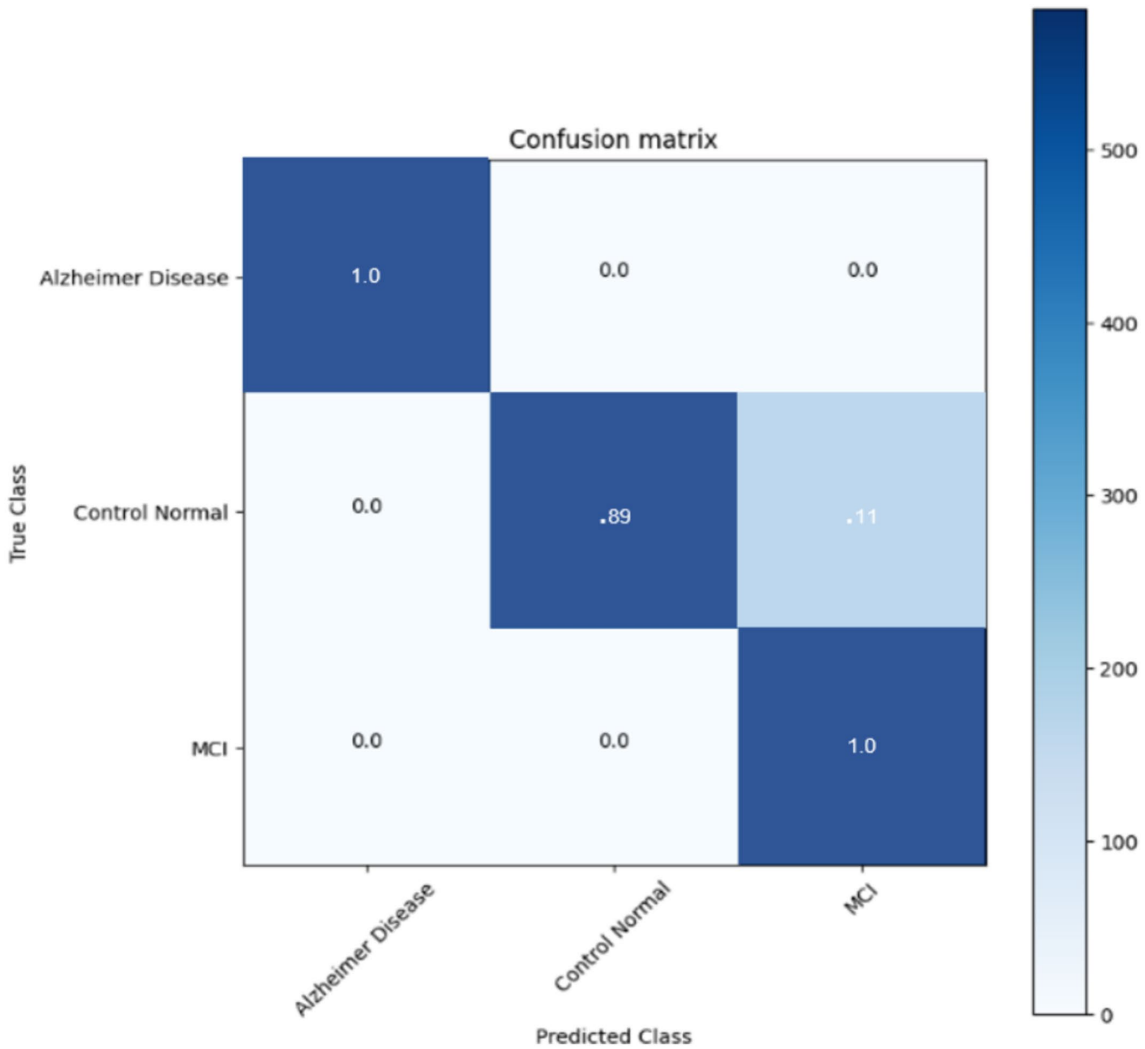
Confusion matrix for DNN CLASSIFIER.

The confusion matrix of the DNN classifier is depicted in the Figure 11 The classifier achieved optimum results with an accuracy of 95.6% in classifying the three stages. The classifier finds incertitude in the classifying stages of MCI and CN. This is because MCI and CN are slightly correlated in the stages, and it is very complex to differentiate. It needs more training and much more detailed differentiation to understand the dissimilarity of the stages.

The comparative analysis of three models SVM, KNN, and HT-DNN demonstrated in the Table 6 shows notable disparities in their categorization skills. The SVM attained an accuracy of 88%, a precision of 88.5%, a recall of 90.3%, and an F1 score of 89.6%. The results demonstrate that although SVM effectively identifies real positive situations, its accuracy is marginally inferior to that of the other models. The SVM exhibits a relatively high recall, indicating it identifies most true positive examples; nevertheless, its precision is inferior to that of other models, implying it may erroneously classify certain instances as positive when they are not. KNN has superior performance, achieving an accuracy of 91.5%, precision of 92%, recall of 91.6%, and an F1 score of 91%. KNN’s balanced metrics indicate superior efficiency in identifying genuine positives and minimizing false positives compared to SVM. Nonetheless, HT-DNN substantially surpasses both models, with an impressive accuracy of 95.6%, precision of 96.3%, recall of 95.4%, and an F1 score of 96%. The HT-DNN model’s superior performance across all metrics indicates it is both more accurate and more reliable for classification tasks, rendering it the optimal choice for applications necessitating precise and balanced predictions, such as in the medical diagnosis of Alzheimer’s disease. To further evaluate the robustness and generalizability of the proposed method, a small-scale validation study was conducted using a held-out subset of unseen MRI scans that were not used during training or cross-validation. The model maintained strong performance, achieving an accuracy of 94.8%, precision of 95.1%, recall of 94.2%, and an F1-score of 94.6%. These results confirm that the proposed hybrid feature extraction and classification approach generalizes well to new data, despite the limited size of the additional validation set. Future work will involve validating the model on larger and more diverse external datasets to further substantiate its clinical applicability.

**Table 6:** Results obtained for the models.

Models	Accuracy	Precision	Recall	F1
SVM	88%	88.5%	90.3%	89.6%
KNN	91.5%	92%	91.6%	91%
HT-DNN	95.6%	96.3%	95.4%	96%

**Figure 12:**
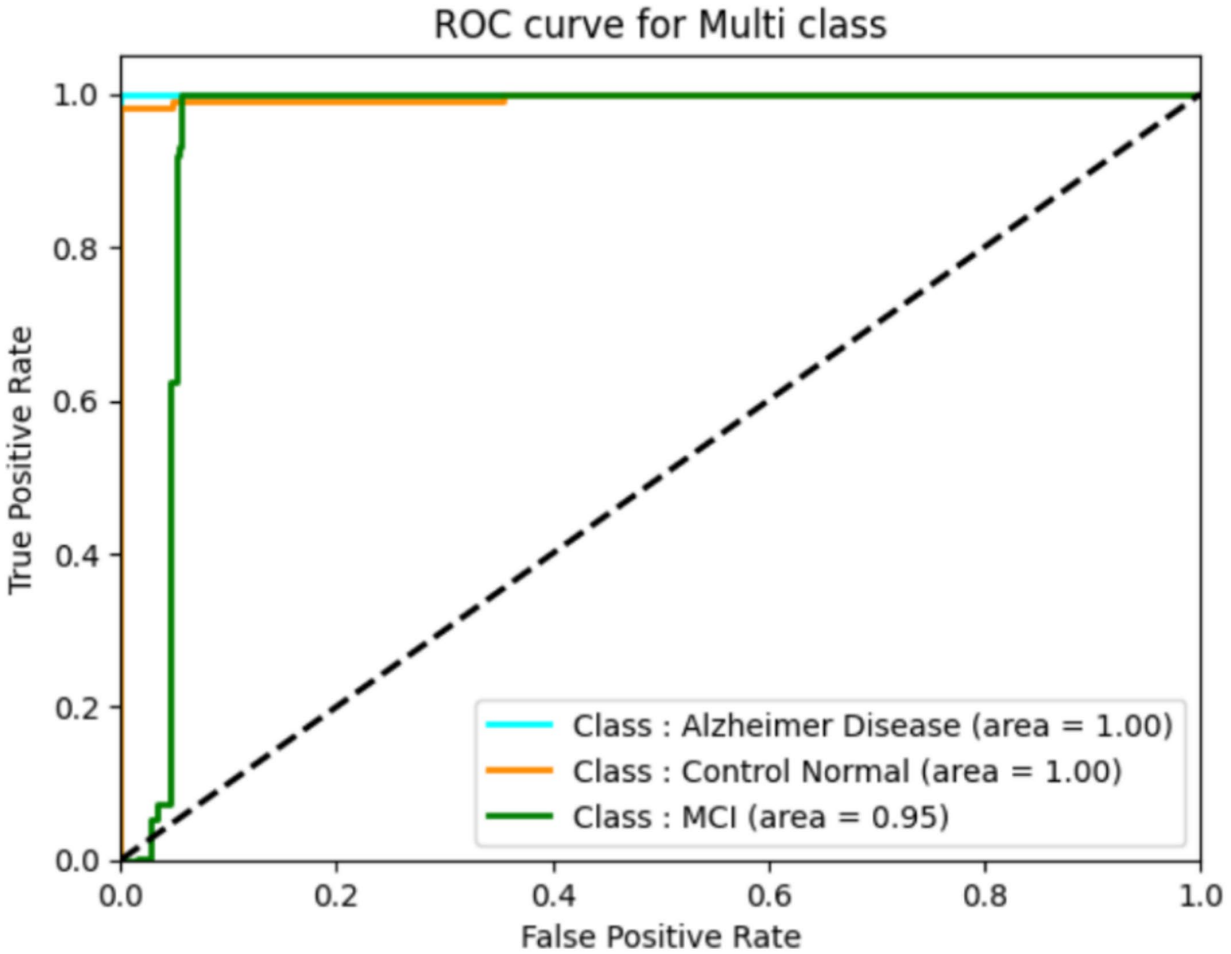
ROC for the DNN model.

The classification performance of a deep learning model can be assessed using ROC curves, particularly in tasks with multi-class. They provide trade-offs between true positive and false positive rates, allowing one to choose the best threshold or gage the model’s effectiveness at various operational points. They provide trade-offs between true positive and false positive rates, allowing one to select the best threshold or gage the model’s effectiveness at different operational points. The following ROC curve (Figure 12) explains the classification of the three classes. We found that our system has a high true fraction value and a low false positive rate when identifying samples from datasets. While the proposed method achieved high classification accuracy, several limitations must be acknowledged. First, the study relies on the ADNI dataset, which, although widely used, may not fully represent the heterogeneity seen in broader clinical settings, especially across different ethnicities, MRI scanners, and acquisition protocols. Second, the handcrafted feature extraction methods (Harris corners and HOG) may limit the ability to capture highly abstract, nonlinear representations compared to end-to-end deep learning models. Additionally, while a 5-fold cross-validation scheme was employed to enhance robustness, external validation on independent datasets is necessary to confirm generalizability. In future work, we plan to integrate multi-modal imaging data (such as PET and fMRI), apply transfer learning strategies, and explore self-supervised learning approaches to improve feature learning and reduce dependence on handcrafted techniques. Expanding the dataset diversity and employing ensemble models may further enhance early diagnosis accuracy and adaptability to real-world clinical applications.

The high accuracy achieved by the proposed model has significant clinical implications. Early and reliable detection of Alzheimer’s Disease, especially at the MCI stage, can enable prompt medical intervention, slow cognitive decline, and improve patient outcomes. Furthermore, automated tools with high precision can support clinicians in early diagnosis, especially in resource-limited settings. The proposed hybrid method demonstrates lower computational complexity compared to full deep learning pipelines. Feature extraction using Harris corners and HOG, followed by classification with SVM, KNN, or DNN, required approximately 4–5 min per MRI volume on an NVIDIA RTX 3060 GPU (12GB VRAM). In contrast, conventional deep CNN-based models often require several hours for training due to larger parameter sets. The reduced processing time and lower hardware demands make the proposed approach more practical for clinical settings, especially where computational resources are limited.

### 6.1 Model interpretability and clinical trust

Interpretability is a critical aspect when applying deep learning models in clinical settings, as clinicians must understand and trust model decisions before adopting them in practice. In the proposed method, interpretability is inherently enhanced through the use of handcrafted features such as Harris corner points and Histogram of Oriented Gradients (HOG). These features correspond to recognizable anatomical landmarks and textural patterns in brain MRI scans, which can be directly correlated with pathological changes associated with Alzheimer’s disease progression. Thus, clinicians can better understand that the model bases its decisions on well-defined and clinically relevant structures rather than abstract representations. Moreover, while this study primarily focused on performance optimization, future enhancements could incorporate visual explanation techniques such as heatmaps, Grad-CAM, or Layer-wise Relevance Propagation (LRP) to highlight regions in the MRI that contribute most to the classification decision. Such visualization methods can further bridge the gap between automated decision-making and clinical reasoning, thereby increasing transparency, building clinician trust, and facilitating adoption in real-world diagnostic workflows. Harris corner detection and HOG extract lower-dimensional, salient features from MRI images before classification, significantly decreasing input data size and network complexity. This handcrafted feature extraction step is far more computationally efficient than training full deep learning models on raw MRI volumes. As a result, the training time for the classifier is reduced from hours (typical for deep CNNs) to just 4–5 min per MRI scan on standard GPU hardware, as observed in our experiments. This efficiency makes the proposed approach more practical and accessible for clinical application, especially in resource-limited settings.

### 6.2 State-of-the-art comparison

In order to elucidate the benefits of the proposed model, a comparative analysis was conducted to assess its efficacy in relation to existing classifiers in the context of brain magnetic resonance (MR) image categorization. Table 7 compares our approach with several state-of-the-art models using accuracy, precision, recall, and F1-score. As shown, our hybrid HOG + DNN method consistently outperforms existing algorithms not only in accuracy but also in terms of balanced sensitivity and specificity. The table clearly illustrates that our current accuracy rate surpasses that of past studies. It is noteworthy to highlight that prior research endeavors have employed deep-learning models for the purpose of addressing diverse multistage Alzheimer’s disease forecasts. The proposed method demonstrates significant improvements over existing state-of-the-art approaches due to its hybrid feature extraction strategy. By integrating Harris corner detection and Histogram of Oriented Gradients (HOG) features prior to classification, the system captures fine-grained local structures and textural patterns critical for differentiating subtle stages of Alzheimer’s Disease. This handcrafted feature extraction enhances noise robustness, particularly in brain MRI scans where image quality variations are common. Unlike purely deep learning-based methods that may require extensive data and are sensitive to noise, the proposed model benefits from stable, transformation-invariant features, leading to better generalization. Quantitatively, our approach achieved an accuracy of 95.6%, outperforming previously reported models that ranged between 85.7 and 94.5%. The improved feature representation and robustness contribute directly to enhanced early-stage detection, enabling more reliable clinical decision-making.

**Table 7:** Performance comparison of existing with proposed method.

Approaches	Dataset	Accuracy	Precision	Recall	F1
Cui et al. (33)	ADNI	85.74%	84.5%	86.0%	85.2%
Zhang et al. (34)	ADNI	88.67%	88.0%	89.0%	88.5%
Liu et al. (35)	ADNI	88.9%	89.1%	88.8%	89.0%
Feng et al. (36)	ADNI	94.52%	95.0%	94.0%	94.5%
Proposed	ADNI	95.6%	96.3%	95.4%	96.0%

### 6.3 Ethical considerations

The use of AI for Alzheimer’s disease diagnosis raises important ethical considerations. Ensuring data privacy is critical, as MRI scans and patient information must be securely stored and handled to protect patient confidentiality. Additionally, while AI models like the one proposed can enhance early detection, there is a risk of misdiagnosis if they are used without proper clinical oversight. Therefore, AI tools should be employed as decision-support systems to assist, rather than replace, expert medical judgment, ensuring that diagnostic outcomes are carefully validated by healthcare professionals.

## 7 Conclusion

The classification of Alzheimer’s disease (AD) is crucial for determining the illness’s stages and guiding suitable treatment strategies. The aforementioned research concentrates on the creation and enhancement of a deep neural network (DNN)-based pipeline, in conjunction with SVM and KNN classifiers, to categorize various phases of Alzheimer’s disease utilizing brain MRI scans. These classifiers have demonstrated commendable accuracy, attaining 91.5% with SVM, 88% with KNN, and an astonishing 95.6% with the DNN model. The superior performance of the DNN pipeline indicates its suitability for addressing the intricate features found in brain pictures, rendering it an excellent instrument for multi-class categorization of Alzheimer’s disease. The accuracy and precision of these models are crucial for assuring effective diagnosis, particularly in differentiating between various phases of cognitive loss. To further confirm the robustness of these classifiers, the research employed ROC curve analysis, a commonly utilized method for assessing the performance of classification models. The ROC curve research validated the efficacy of the suggested pipeline, establishing that it provides a more dependable classification framework than other established classifiers. The study shown, through comparison analysis, which the DNN excels in accurately identifying Alzheimer’s stages across different age groups, underscoring the need of incorporating this framework into future diagnostic methodologies. As research advances, the use of sophisticated data mining techniques and the consolidation of many datasets are expected to enhance the model’s efficacy, potentially facilitating earlier diagnosis of Alzheimer’s disease and better treatment results. These initiatives will concentrate on utilizing multi-modal data to improve predictive accuracy, striving for more precise and earlier-stage Alzheimer’s forecasts among various groups. Future work should extend validation to larger, more diverse, and real-time patient datasets across different clinical centers and imaging protocols. This will strengthen confidence in the model’s generalizability and clinical reliability. Combining structural MRI features with other biomarkers (e.g., PET, CSF biomarkers, genetic data) has the potential to further improve diagnostic accuracy and offer a more holistic assessment of neurodegeneration. AI models developed from robust feature extraction and classification frameworks can facilitate individualized risk scoring and prognosis, enabling tailored therapeutic planning and monitoring of high-risk individuals.

## Data Availability

The original contributions presented in the study are included in the article/supplementary material, further inquiries can be directed to the corresponding author.
